# Monophonic and Polyphonic Wheezing Classification Based on Constrained Low-Rank Non-Negative Matrix Factorization

**DOI:** 10.3390/s21051661

**Published:** 2021-02-28

**Authors:** Juan De La Torre Cruz, Francisco Jesús Cañadas Quesada, Nicolás Ruiz Reyes, Sebastián García Galán, Julio José Carabias Orti, Gerardo Peréz Chica

**Affiliations:** 1 Department of Telecommunication Engineering, University of Jaen, Campus Cientifico-Tecnologico de Linares, Avda. de la Universidad, s/n, Linares, 23700 Jaen, Spain; fcanadas@ujaen.es (F.J.C.Q.); nicolas@ujaen.es (N.R.R.); sgalan@ujaen.es (S.G.G.); carabias@ujaen.es (J.J.C.O.); 2 Pneumology Clinical Management Unit of the University Hospital of Jaen, Av. del Ejercito Espanol, 10, 23007 Jaen, Spain; gerardo.perez.sspa@juntadeandalucia.es

**Keywords:** monophonic, polyphonic, wheezing, non-negative matrix factorization, spectral pattern, spectrogram, constraint, low-rank, asthma, chronic obstructive pulmonary disease

## Abstract

The appearance of wheezing sounds is widely considered by physicians as a key indicator to detect early pulmonary disorders or even the severity associated with respiratory diseases, as occurs in the case of asthma and chronic obstructive pulmonary disease. From a physician’s point of view, monophonic and polyphonic wheezing classification is still a challenging topic in biomedical signal processing since both types of wheezes are sinusoidal in nature. Unlike most of the classification algorithms in which interference caused by normal respiratory sounds is not addressed in depth, our first contribution proposes a novel Constrained Low-Rank Non-negative Matrix Factorization (CL-RNMF) approach, never applied to classification of wheezing as far as the authors’ knowledge, which incorporates several constraints (sparseness and smoothness) and a low-rank configuration to extract the wheezing spectral content, minimizing the acoustic interference from normal respiratory sounds. The second contribution automatically analyzes the harmonic structure of the energy distribution associated with the estimated wheezing spectrogram to classify the type of wheezing. Experimental results report that: (i) the proposed method outperforms the most recent and relevant state-of-the-art wheezing classification method by approximately 8% in accuracy; (ii) unlike state-of-the-art methods based on classifiers, the proposed method uses an unsupervised approach that does not require any training.

## 1. Introduction

Chronic Respiratory Diseases (CRDs) are increasingly a huge and growing public health problem due to their high prevalence, high morbidity and mortality, and socio-economic cost. CRDs can be defined as disorders of the airways and other physiological structures of the respiratory system [1]. Some of the most common and relevant CRDs are asthma and Chronic Obstructive Pulmonary Disease (COPD). According to the World Health Organization (WHO), there were 417,918 deaths due to asthma at the global level in 2016 [2] and approximately three million people die from COPD every year, which is 6% of all deaths worldwide [3]. Although chronic diseases currently have no medical cure, early detection can lead to appropriate treatment when the disease is in its early stages, thus improving people’s quality of life.

The auscultation examination is considered a widely used method of detecting CRDs because it is a non-invasive, inexpensive, easy, comfortable, and fast method regardless of age [4]. However, the auscultation process has several limitations that reduce the reliability of the diagnosis: (i) high subjectivity conditioned by the physician’s training to recognize and interpret the sounds captured by the stethoscope [5,6]; (ii) the discrimination between adventitious sounds with similar characteristics, such as monophonic and polyphonic wheezing sounds, is a harder task to perform by means of auscultation [7]; and (iii) normal respiratory sounds and adventitious sounds (abnormal and indicative of a lung disorder) are simultaneously mixed in the time and frequency domain, complicating the physician’s analysis of the valuable clinical information contained in adventitious sounds [5,8,9]. Considering the above, a misdiagnosis is the main cause of the patient returning to the health center with a worsening of the disease that was not detected in the first medical examination performed by auscultation, so in recent years, it has become crucial to develop novel approaches to help physicians provide reliable diagnoses applied to lung disorders, with the implicit fact of reducing health care costs [10,11].

In general, the sounds generated during breathing can be classified into two main categories: normal respiratory sounds and adventitious sounds. Both sounds are mixed in the time-frequency domain as they are simultaneously generated by the same air flow through the bronchial tree of the lungs and also share part of the spectral bands in which they are active [5,9]. Normal respiratory sounds are represented by a wideband spectrum where most of the energy is concentrated in the frequency band of 60 Hz–1000 Hz [12]. Adventitious sounds can be classified into two categories: discontinuous and continuous sounds. Discontinuous Adventitious Sounds (DASs) are characterized by a short duration of less than 25 ms, such as coarse and fine crackles [13]. Continuous Adventitious Sounds (CASs) are characterized by a long duration of more than 100 ms, such as rhonchi, stridor, and wheezing [14]. In recent years, several works have been published that carried out an exhaustive review of lung acoustic measurements [15] and signal processing methods [16,17] applied to adventitious sounds, most of them focused on detection [16,17,18,19,20,21,22,23] and classification tasks [16,17,24,25,26,27,28,29].

Focusing on wheezing or wheeze sounds, the guidelines established by Computerized Respiratory Sound Analysis (CORSA) [5,30] define them as a pitch located between 100 Hz and 1000 Hz whose duration is greater than 100 ms, displaying trajectories of narrowband spectral peaks over time. The appearance of wheezing is widely considered by doctors as a clue to be able to detect either respiratory diseases or the severity associated with CRDs early, as occurs in the case of asthma and COPD [31,32]. For this reason, many research efforts have been applied in biomedical signal processing in order to develop reliable methods for early wheezing detection. In this sense, many wheezing detection algorithms, based on different approaches, can be found in the state-of-the-art literature: the Autoregressive (AR) model [33], auditory modeling [34], entropy [35], Neural Networks (NN) [36,37], wavelet transform [38,39], tonal index [40,41], Mel-Frequency Cepstral Coefficients (MFCCs) [42,43], Gaussian Mixture Models (GMMs) [44,45], spectral peaks identification [46,47,48], the Hidden Markov Model (HMM) [49], and recently, Non-negative Matrix Factorization (NMF) [9,50,51].

**Figure 1:**
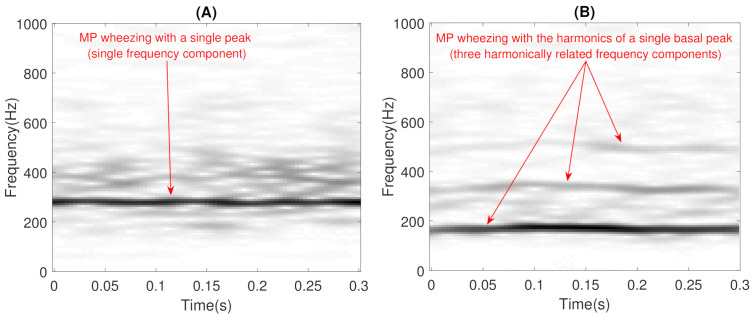
Time-frequency representation of two examples of Monophonic (MP) wheezing: (**A**) with a single basal peak; (**B**) with the harmonics of a single basal peak. Note that the frequency components are harmonically related in (**B**).

**Figure 2:**
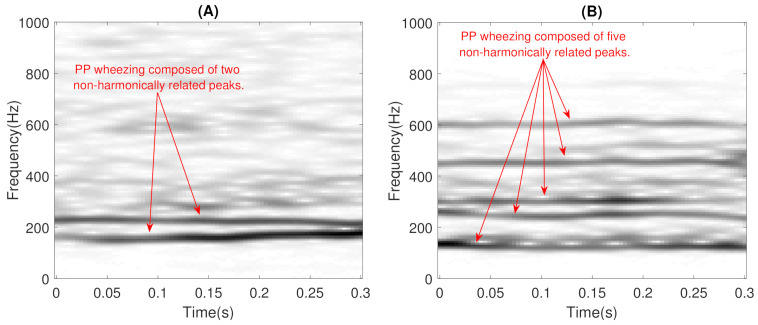
Time-frequency representation of two examples of Polyphonic (PP) wheezing: (**A**) with two non-harmonically related peaks; (**B**) with five non-harmonically related peaks. Note that the frequency components are not harmonically related in the case of PP wheezing.

In addition, wheezing can be classified into two main categories according to the spectral behavior [52]: (i) wheezes that occur with a single peak or with the harmonics associated with that single basal peak are called Monophonic (MP) wheezes (as can be seen in Figure 1); and (ii) wheezes that occur with variable peaks that differ in harmonics are called Polyphonic (PP) wheezes (as can be seen in Figure 2). The scientific interest in the field of biomedical sound signal processing in automatically performing this classification lies in the fact that PP wheezes are usually caused by the pathology of small airways and MP wheezes are caused by the pathology of larger airways [53]. In fact, several studies [4,54,55,56] have shown that MP and PP wheezes exhibit distinctive physiological and pathological characteristics: (i) in physiological analysis, MP wheezes are caused by a single bronchial narrowing, while PP wheezes are caused by multiple central bronchial compression; and (ii) in pathological analysis, MP wheezes are an indicator of the presence of asthma, while PP wheezes can be considered as a sound marker of COPD.

Despite advances in the analysis of respiratory sounds, MP/PP wheezing classification is a critical step in the diagnosis of asthma [4,54,55] and COPD diseases [54,55,56], so it is still a challenging topic in biomedical signal processing [7] since both types of wheezes are sinusoidal in nature. Although there are relatively few works [7,18,47,57,58,59,60] in which the analysis of MP/PP wheezing is treated, the only works focused on the task of classifying MP/PP wheezing in depth are [7,57,59,60] to our knowledge. All these MP/PP wheezing classification approaches are based on the feature extraction and classifier configuration. Ulukaya et al. [7] proposed to extract a single feature, the Peak Energy Ratio (PER), from a RAtional Dilation Wavelet Transform (RADWT) to discriminate between MP and PP wheezes. Specifically, PER is obtained from the first and second peak with the highest energy of all sub-bands of the wavelet coefficients (considering that the second peak is not consecutive to the first one). Moreover, the authors applied a robust evaluation methodology in which most of the relevant feature extraction methods [57,59,60] were evaluated using some of the most popular classifiers (SVM, KNN, and ELM) and Leave-One-Out (LOO) cross-validation schemes. The results reported that the proposed method, based on only one feature (PER), obtained the best MP/PP wheezing classification performance showing an accuracy equal to 86%.

However, none of the state-of-the-art methods consider the interference generated by normal respiratory sounds that can affect the MP/PP wheezing classification task. In this work, our proposal is based on the Non-negative Matrix Factorization (NMF) approach in order to classify MP/PP wheezing sounds according to the harmonic structure shown by removing the sound interference caused by normal respiratory sounds. The first contribution of this work proposes a novel Constrained Low-Rank Non-negative Matrix Factorization (CL-RNMF) approach, which allows the spectral patterns associated with wheezing sounds to be extracted with the least possible sound interference from normal breath sounds. Specifically, we propose a low-rank configuration using a reduced number of wheeze bases to compact the frequency components into the fewest possible bases for further analysis without loss of relevant wheeze content. In addition, the proposed CL-RNMF approach incorporates a set of constraints to model the spectro-temporal behavior of wheezing and normal respiratory sounds. These constraints help to acoustically isolate the wheezing spectral patterns from normal respiratory sounds. To classify between MP or PP wheezing sounds, the second contribution analyzes the harmonic structure of the previous reduced number of wheezing bases based on the spectral location of the wheezing components, rather than the energy of their components.

The structure of the paper is as follows. Section 2 briefly reviews the principles of non-negative matrix factorization, focusing on the standard approach and some regularizations used to model the properties of the sounds of interest. The proposed MP/PP wheezing classification method is presented in Section 3. Section 4 details and discusses the experimental evaluation. Finally, we conclude in Section 5 and provide perspectives on further research.

## 2. Theoretical Background

### 2.1. Non-Negative Matrix Factorization

Non-negative Matrix Factorization (NMF) or standard NMF [61,62] is a decomposition technique that has attracted special attention in different fields of biomedical signal processing in the last few years [63,64]. Previous works showed the efficiency of the NMF approach at detecting [9,50,51] and improving the audio quality of wheezing [65,66]. In general terms, NMF can be defined as an unsupervised learning tool used for linear representation of non-negative two-Dimensional (2D) data where its main advantage is to reduce the dimensionality of a large amount of data in order to find hidden structures by means of part-based representation with non-negative patterns. From a mixture signal x(t), its magnitude spectrogram $X\in R_{+}^{F\times T}$ is obtained by means of the Short-Time Fourier Transform (STFT) applying a window function (e.g., Hamming or Hann) and inter-window overlap to increase the temporal resolution, *F* being the number of frequency bins and *T* the number of time frames. Here, standard NMF decomposes the magnitude spectrogram X into the product of two non-negative matrices: spectral basis matrix (patterns) $B\in R_{+}^{F\times K}$ and temporal activation matrix (weights) $A\in R_{+}^{K\times T}$, *K* being the rank or the number of components (spectral bases),

(1) $$X\approx \hat{X}=BA$$

where $\hat{X}\in R_{+}^{F\times T}$ is the estimated spectrogram. Each column of the basis matrix B defines a spectral pattern that describes the spectral behavior of an active sound event in the input spectrogram X. Each row of the activation matrix A represents a temporal gain for a spectral pattern. In other words, the matrix B provides a dictionary composed of *K* spectral bases, and the matrix A defines the weight with which the different spectral bases appear along the temporal frames. Due to the non-negativity property, NMF underlies an additive linear interpolation model that results in the so-called part-based representation [61].

The decomposition or factorization of the input magnitude spectrogram X into the product BA is usually sought minimizing a defined scalar-valued divergence,

(2) $$\arg\min_{B,A}D\left(X|BA\right)\;B,A\geq 0$$

This divergence function measures the error made in the approximation of the observed spectrogram X and the reconstruction BA. Typically, the divergence is computed entry-wise:

(3) $$D\left(X|\hat{X}\right)=D\left(X|\mathrm{BA}\right)=\sum_{f=1}^{F}\sum_{t=1}^{T}d(X_{f,t}|{\hat{X}}_{f,t})$$

where d(i,j) is a function of two scalar variables i,j. It is often called the cost function and is a positive function of $i\in R_{+}$ given $j\in R_{+}$ with a single minimum for i=j. Some of the most popular cost functions are the Euclidean distance, the generalized Kullback–Leibler divergence, the Itakura–Saito divergence, and the Cauchy distribution [67,68]. In this paper, we propose to minimize the generalized Kullback–Leibler divergence $D_{KL}\left(X|\hat{X}\right)$ (see Equation (4)) because previous works [9,50,51,63,65,66] obtained promising results in biomedical signal processing since $D_{KL}\left(X|\hat{X}\right)$ provides a scale-invariant factorization, that is low energy sound components of X bear the same relative importance as high energy ones in the decomposition process.

(4) $$D_{KL}\left(X|\hat{X}\right)=\sum_{f=1}^{F}\sum_{t=1}^{T}X_{f,t}\log\frac{X_{f,t}}{{\hat{X}}_{f,t}}-X_{f,t}+{\hat{X}}_{f,t}$$

The most popular minimization method to solve the problem shown in Equation (2) is based on the so-called multiplicative update rules, initially proposed by Lee and Seung [61]. This method obtains the basis and activation matrices, minimizing the Kullback–Leibler divergence function $D_{KL}\left(X|\hat{X}\right)$ and ensuring the non-negativity of the estimated matrices. These rules are obtained directly from the negative and positive terms of the partial derivative of the divergence function $D_{KL}\left(X|\hat{X}\right)$ with respect to the parameters B and A,

(5) $$B\leftarrow B⊙\frac{{\left[\frac{\partial D_{KL}\left(X|\hat{X}\right)}{\partial B}\right]}^{-}}{{\left[\frac{\partial D_{KL}\left(X|\hat{X}\right)}{\partial B}\right]}^{+}}=B⊙\left(\left(X⊘\mathrm{BA}\right)A^{T}⊘\left(\left[1\right]A^{T}\right)\right)$$

(6) $$A\leftarrow A⊙\frac{{\left[\frac{\partial D_{KL}\left(X|\hat{X}\right)}{\partial A}\right]}^{-}}{{\left[\frac{\partial D_{KL}\left(X|\hat{X}\right)}{\partial A}\right]}^{+}}=A⊙\left(B^{T}\left(X⊘\mathrm{BA}\right)⊘\left(B^{T}\left[1\right]\right)\right)$$

where $\left[1\right]\in R_{+}^{F\times T}$ represents an all-ones matrix, ${}^{T}$ is the transpose operator, ⊙ is the element-wise multiplication, and ⊘ is the element-wise division. This procedure always maintains the non-negativity of both parameters, since the used terms in the updating are also non-negative.

As previously described, NMF models the magnitude spectrogram of an input mixture signal as a product of a basis matrix and an activation matrix with the only constraint of the element-wise non-negativity of all matrices. Under this constraint, the aim is to minimize the cost function of the reconstruction error. However, the main problem of NMF is the trade-off between signal reconstruction and the physical interpretation of the factorized part-based objects. In other words, this non-negativity of the parameters does not guarantee a meaningful part-based representation when dealing with real-world mixture signals [69,70]. Several properties can be used to improve the uniqueness of the local minima obtained by NMF, incorporating physical meaning into the basis functions and activations. In particular, these properties can be implemented using regularizations, which are added to the global cost function in the factorization model. The main constraints, sparseness and smoothness, used in this paper to model the spectro-temporal behavior of wheezing and normal respiratory sounds are briefly described below.

### 2.2. Spectral Sparseness

Spectral sparseness ψ(B) denotes that, for each source, most of its frequencies are zero or close to zero [71,72]. This constraint enforces that only a few frequency bins predominate in each spectral basis, whilst the other bins are canceled. It is implemented by incorporating a penalty term into the NMF objective function. In practice, the $L^{1}$-norm is often used because it was demonstrated to be less sensitive to changes of the parameter that controls the importance of the constraint in the factorization process. Then, the optimization problem can be expressed as,

(7) $$\arg\min_{B,A}D\left(X|BA\right)+\alpha {∥B∥}_{1}\;B,A\geq 0$$

where α is the weight parameter that adjusts the influence of the constraint.

### 2.3. Temporal/Spectral Smoothness

Generally, smoothness ϕ means how continuous or smooth the spectral or temporal changes related to a source are [72]. Smoothness constraints have been defined for both activation A and basis functions B and added to the global cost function as penalty terms as follows,

(8) $$\arg\min_{B,A}D\left(X|BA\right)+\lambda \phi \left(A\right)+\beta \phi \left(B\right)\;B,A\geq 0$$

where ϕ(A) and ϕ(B) are the functions that penalize non-smooth temporal activations or spectral patterns and the parameters λ and β control the effect of the regularization in the decomposition procedure.

Temporal smoothness (also known as smooth activations) ϕ(A), applied to the estimated activation matrix A, reports how slow the amplitude variations over time are. In other words, temporal smoothness accounts for the fact that real-world sounds usually have a temporal structure, and their acoustic characteristics vary slowly as a function of time. In [72], the authors proposed to model the temporal smoothness regularization ϕ(A) by applying a high cost to large changes produced between adjacent frames in the activation matrix A as follows,

(9) $$\phi \left(A\right)=\sum_{k=1}^{K}\frac{1}{\sigma_{k}^{2}}\sum_{t=2}^{T}{\left(A_{k,t}-A_{k,t-1}\right)}^{2}$$

where $\sigma_{k}=\sqrt{\frac{1}{T}\sum_{t=1}^{T}A_{k,t}^{2}}$ indicates the standard deviation used to normalize the activation functions. This normalization provides that the cost of regularization is independent of the numerical scale of activation [69,72].

Spectral smoothness (also known as smooth basis) ϕ(B), applied to the estimated basis matrix B, measures how fast the amplitude changes along the frequency axis, that is it allows modeling the behavior of those sounds that are represented by a wideband spectrum. In [69,73], the authors proposed to model the spectral smoothness regularization ϕ(B) by applying a high cost to large changes produced between adjacent bins in the basis matrix B as follows,

(10) $$\phi \left(B\right)=\sum_{k=1}^{K}\frac{1}{\sigma_{k}^{2}}\sum_{f=2}^{F}{\left(B_{f,k}-B_{f-1,k}\right)}^{2}$$

where $\sigma_{k}=\sqrt{\frac{1}{F}\sum_{f=1}^{F}B_{f,k}^{2}}$ represents the standard deviation used to normalize the basis functions. This normalization achieves that the cost of regularization is independent of the numerical scale of the basis [69,73].

## 3. Proposed Method

**Figure 3:**
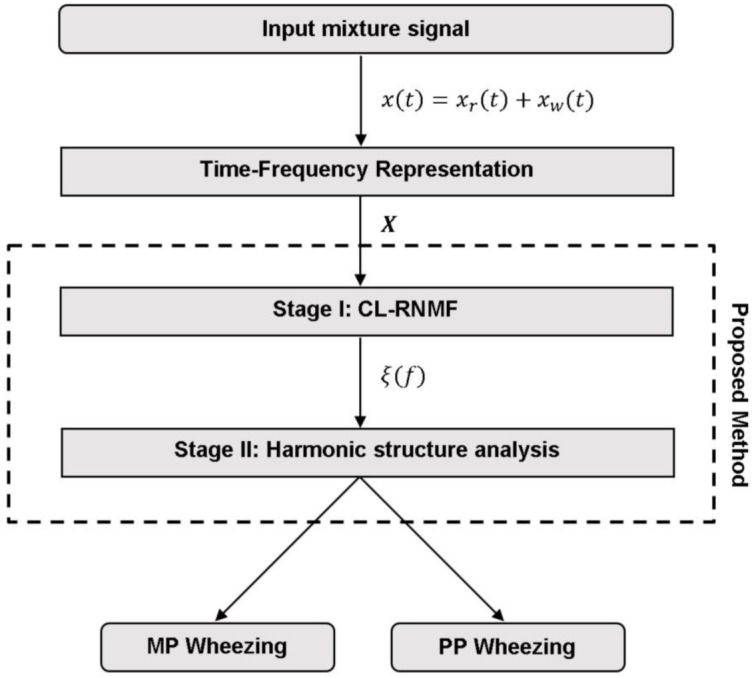
Flowchart of the proposed method.

The main problem in classifying wheezes from a mixture is that both wheezing sounds and normal respiratory sounds occur simultaneously in the time and frequency domain. Considering the acoustic interference caused by normal respiratory sounds, the proposed signal model is composed of two stages: Modelling and separation of wheezing spectral patterns from normal respiratory sounds based on CL-RNMF (stage I) and Classification between MP/PP wheezing according to its harmonic structure (stage II). In this manner, the goal of the stage I is to model the spectral patterns that characterize wheezing sounds by isolating them from respiratory interference. The aim of stage II is to analyze the location of the frequency components extracted from the previous stage to determine the type, monophonic or polyphonic, of wheezing according how the wheezing energy is locating in the frequency domain. The flowchart of the proposed method is shown in Figure 3, and details are depicted in Section 3.1, Section 3.2 and Section 3.3.

### 3.1. Time-Frequency Signal Representation

Time-frequency representation by means of spectrograms has been demonstrated to be useful for visualizing the characteristics and behavior of both wheezing and normal respiratory sounds [9,50,51,65,66]. The input mixture signal x(t) is composed of wheeze sounds $x_{w}\left(t\right)$ (MP or PP wheezing) and normal respiratory sounds $x_{r}\left(t\right)$ overlapping in the time and frequency domain. We assume that the mixture of these sounds is additive and can be expressed as $x\left(t\right)=x_{r}\left(t\right)+x_{w}\left(t\right)$. The input magnitude spectrogram $X\in R_{+}^{F\times T}$ of the input mixture signal can be represented as $X=X_{R}+X_{W}$, being $X_{R}\in R_{+}^{F\times T}$ the magnitude spectrogram of only respiratory sounds and $X_{W}\in R_{+}^{F\times T}$ the magnitude spectrogram of only wheeze sounds. Specifically, each magnitude spectrogram is composed of *T* frames, *F* frequency bins and a set of time-frequency units $X_{f,t}$, being f=1,…,F and t=1,…,T. Each unit $X_{f,t}$ is defined by the *f*th frequency bin at the *t*th frame and is calculated from the magnitude of the Short-Time Fourier Transform (STFT) using a Hamming windows of *N* samples with 10% overlap. In this work, a normalization process is applied in order to achieve independence regarding the size and scale of the input spectrogram X. Thus, the normalized magnitude spectrogram $\bar{X}$ is computed as follows,

(11) $$\bar{X}=\frac{X}{\left(\frac{\sum_{f=1}^{F}\sum_{t=1}^{T}X_{f,t}}{FT}\right)}$$

To avoid complex nomenclature throughout the paper, the variable X is hereinafter referred to the normalized magnitude spectrogram previously computed in Equation (11).

### 3.2. Stage I: Constrained Low-Rank Non-Negative Matrix Factorization

As mentioned above, it is common that normal respiratory sounds mask the presence of the wheezing sounds. As a result, this sound mask makes the task of wheezing classification difficult since the spectral patterns associated with normal respiratory sounds can be confused with wheezing spectral content. Therefore, the aim of this stage is to provide a reliable modeling of the different frequency components (spectral patterns) that compose a wheeze, removing any sound interference from normal respiratory sounds. For this purpose, we propose a CL-RNMF approach because, as far as the authors’ knowledge, the non-negative matrix factorization approach has never been applied before to MP/PP wheezing classification. In addition, our approach is an unsupervised method because it does not require any training of the sounds to classify. Therefore, the proposed method decomposes a magnitude mixture spectrogram X into two estimated spectrograms: ${\hat{X}}_{R}$ (only normal respiratory sounds without wheezing) and ${\hat{X}}_{W}$ (only wheeze sounds without normal respiratory sounds). In this manner, each estimated spectrogram can be factorized into the product of its corresponding estimated basis and activation matrices: (i) $B_{R}\in R_{+}^{F\times K_{r}}$ and $A_{R}\in R_{+}^{K_{r}\times T}$ to the factorization of ${\hat{X}}_{R}$, $K_{r}$ being the number of respiratory components; and (ii) $B_{W}\in R_{+}^{F\times K_{w}}$ and $A_{W}\in R_{+}^{K_{w}\times T}$ to the factorization of ${\hat{X}}_{W}$, $K_{w}$ being the number of wheezing components. The proposed separation model can be formulated with the following objective function,

(12) $$X\approx {\hat{X}}_{R}+{\hat{X}}_{W}=B_{R}A_{R}+B_{W}A_{W}$$

where, considering the non-negative property that characterizes the NMF approach, all the matrices that compose the previous model are non-negative.

As previously mentioned, this stage attempts to ensure that $B_{W}$ contains reliable modeling of the wheezing spectral patterns by means of narrowband spectral peaks that typically characterize the wheeze content. The key assumptions behind the proposed CL-RNMF approach to model wheezing spectral patterns are the following:

- **Low-rank**: The number of wheezing components should be much less than the number of normal respiratory components, that is $K_{w}\ll K_{r}$. This assumption allows that the number of frequency components can be reduced in the least number of bases possible for their posterior analysis, while normal respiratory sounds are modeled using a higher range of components. Experimental results showed that the best classification performance was obtained when $2\leq K_{w}\leq 6$ and $K_{r}\geq 32$. In particular, when $K_{w}=1$, the proposed CL-RNMF approach tends to converge very quickly at the expense of losing relevant wheezing content. On the other hand, when $K_{w}>6$, the spectral wheezing patterns tend to be split into different components of the matrix $B_{W}$.
- **Constraints**: These characterize wheezing sounds and normal respiratory sounds using opposite restrictions between both sounds. The use of constraints allows isolating the spectral wheezing patterns from the spectral patterns of normal respiratory sounds. Therefore, in order to find a better NMF decomposition that shows spectro-temporal features of the wheezing and normal respiratory sounds as can be observed in the real world, we propose to incorporate sparseness and smoothness into the NMF decomposition process. As shown in Figure 1 and Figure 2, wheezing sounds can be considered sparse in frequency because MP wheezing or PP wheezing is characterized by one or more than one narrowband spectral peak. Moreover, wheezing sounds can be considered smooth or continuous events in time, that is slow variation of the magnitude spectrogram along time. On the other hand, normal respiratory sounds can be considered smooth in frequency, that is they can be modeled assuming wideband spectral patterns. Therefore, $B_{W}$ should contain wheezing spectral patterns composed of one or more than one narrowband spectral peak, depending on the spectral complexity of each wheezing, and $B_{R}$ should be composed of a set of wideband spectral patterns that model the behavior of normal respiratory sounds.

Considering the key assumptions mentioned above, the global objective function $D\left(X|\hat{X}\right)$ that must be minimized in order to estimate the basis ($B_{R}$, $B_{W}$) and activation ($A_{R}$, $A_{W}$) matrices is composed of: (i) the Kullback–Leibler divergence cost function $D_{KL}\left(X|\hat{X}\right)$ to minimize the reconstruction error between the input spectrogram X and the estimated spectrogram $\hat{X}$, (ii) the spectral sparseness $\psi \left(B_{W}\right)$ and temporal smoothness $\phi \left(A_{W}\right)$ restrictions applied to $B_{W}$ and $A_{W}$, respectively, to model the wheezing spectral patterns, and (iii) the spectral smoothness $\phi \left(B_{R}\right)$ restriction applied to $B_{R}$, to model the spectral patterns of normal respiratory sounds. The global objective function $D\left(X|\hat{X}\right)$ is detailed as follows,

(13) $$D\left(X|\hat{X}\right)=D_{KL}\left(X|\hat{X}\right)+\alpha \psi \left(B_{W}\right)+\lambda \phi \left(A_{W}\right)+\beta \phi \left(B_{R}\right)$$

where the equations of terms $D_{KL}\left(X|\hat{X}\right)$, $\psi \left(B_{W}\right)$, $\phi \left(A_{W}\right)$, and $\phi \left(B_{R}\right)$ can be found in Section 2. The parameters α, λ, and β define the weight to control the effect of the regularization. Experimental results showed that the best classification performance is obtained when all weights are equal α=λ=β, the optimal value being α=λ=β=0.5. Analyzing the sound separation performance of the previous decomposition, we observed empirically that the acoustic interference suffered by wheezing sounds from normal respiratory sounds is minimum, and no significant loss of wheezing content occurs when α=λ=β. However, significant losses of wheezing content appear when α=λ>β, or significant sound interference by normal respiratory sounds can be observed when α=λ<β.

**Figure 4:**
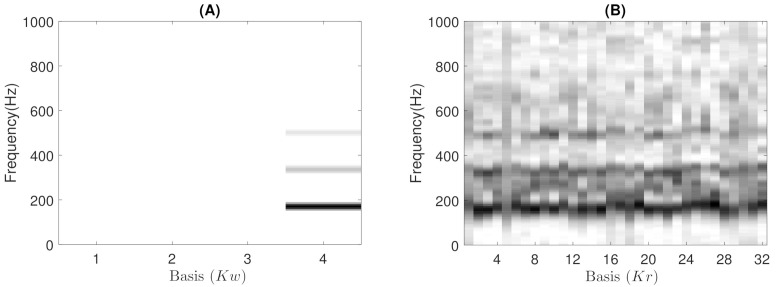
Example of the estimated matrices $B_{W}$ and $B_{R}$ obtained from the proposed CL-RNMF approach, analyzing the MP wheezing spectrogram previously shown in Figure 1B. (**A**) Although the matrix $B_{W}$ is composed of four spectral bases, the spectral wheezing patterns are compacted into the fourth basis $B_{W}\left(4\right)$. This spectral basis $B_{W}\left(4\right)$ is composed of three narrowband spectral peaks. (**B**) The matrix $B_{R}$ is composed of thirty-two wideband spectral bases.

From Equation (13), the estimated basis matrices ($B_{W}$ and $B_{R}$) and activation matrices ($A_{W}$ and $A_{R}$) can be obtained by applying a gradient descent algorithm based on multiplicative update rules. Specifically, the multiplicative update rules to learn those matrices can be computed by taking negative and positive terms of the partial derivative of the global objective function $D\left(X|\hat{X}\right)$ with respect to $B_{W}$, $B_{R}$, $A_{W}$, and $A_{R}$, respectively,

(14) $$B_{W}\leftarrow B_{W}⊙\frac{{\left[\frac{\partial D_{KL}\left(X|\hat{X}\right)}{\partial B_{W}}\right]}^{-}+\alpha {\left[\frac{\partial \psi (B_{W})}{\partial B_{W}}\right]}^{-}}{{\left[\frac{\partial D_{KL}\left(X|\hat{X}\right)}{\partial B_{W}}\right]}^{+}+\alpha {\left[\frac{\partial \psi (B_{W})}{\partial B_{W}}\right]}^{+}}$$

(15) $$B_{R}\leftarrow B_{R}⊙\frac{{\left[\frac{\partial D_{KL}\left(X|\hat{X}\right)}{\partial B_{R}}\right]}^{-}+\beta {\left[\frac{\partial \phi (B_{R})}{\partial B_{R}}\right]}^{-}}{{\left[\frac{\partial D_{KL}\left(X|\hat{X}\right)}{\partial B_{R}}\right]}^{+}+\beta {\left[\frac{\partial \phi (B_{R})}{\partial B_{R}}\right]}^{+}}$$

(16) $$A_{W}\leftarrow A_{W}⊙\frac{{\left[\frac{\partial D_{KL}\left(X|\hat{X}\right)}{\partial A_{W}}\right]}^{-}+\lambda {\left[\frac{\partial \phi (A_{W})}{\partial A_{W}}\right]}^{-}}{{\left[\frac{\partial D_{KL}\left(X|\hat{X}\right)}{\partial A_{W}}\right]}^{+}+\lambda {\left[\frac{\partial \phi (A_{W})}{\partial A_{W}}\right]}^{+}}$$

(17) $$A_{R}\leftarrow A_{R}⊙\frac{{\left[\frac{\partial D_{KL}\left(X|\hat{X}\right)}{\partial A_{R}}\right]}^{-}}{{\left[\frac{\partial D_{KL}\left(X|\hat{X}\right)}{\partial A_{R}}\right]}^{+}}$$

where, for each multiplicative update rule, the division between the negative and positive terms of the partial derivatives is an element-wise division. More details related to the equations of each partial derivative of the multiplication update rules can be found in Appendix A. Finally, the estimated respiratory and wheezing basis ($B_{W}$ and $B_{R}$) and activation matrices ($A_{W}$ and $A_{R}$) are obtained updating the previous rules until the algorithm converges using *M* iterations. Figure 4 shows the estimated matrices $B_{W}$ and $B_{R}$ decomposing the MP wheezing spectrogram shown in Figure 1B. As can be observed, the matrix $B_{W}$ contains spectral patterns that characterize a typical MP wheezing, which are represented by means of a set of narrowband spectral peaks (or frequency components). In contrast, the estimated matrix $B_{R}$ is composed of a set of wideband spectral patterns that characterize normal respiratory sounds. Therefore, the proposed CL-RNMF approach achieves extracting the wheezing spectral content at the expense of removing normal respiratory sounds.

**Figure 5:**
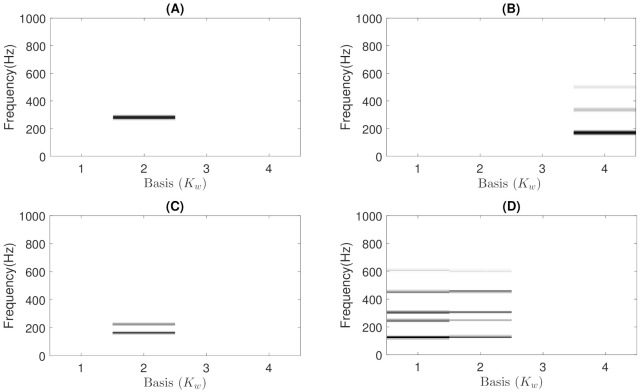
The estimated basis matrices $B_{W}$ obtained from CL-RNMF in the examples shown in Section 1. (**A**) $B_{W}$ for the MP wheezing shown in Figure 1A. (**B**) $B_{W}$ for the MP wheezing shown in Figure 1B. (**C**) $B_{W}$ for the PP wheezing shown in Figure 2A. (**D**) $B_{W}$ for the PP wheezing shown in Figure 2B. The wheezing spectral patterns were compacted into a single basis, $B_{W}\left(2\right)$ (in Case (**A**)), $B_{W}\left(4\right)$ (in Case (**B**)), and $B_{W}\left(2\right)$ (in Case (**C**)). However, the energy of the narrowband spectral peaks was divided into two bases $B_{W}\left(1\right)$ and $B_{W}\left(2\right)$, as can be seen in Case (**D**).

Experimentally, we found that the proposed CL-RNMF approach tends to compact all the narrowband spectral peaks into a single basis of the matrix $B_{W}$, as shown in Figure 4A. However, considering that CL-RNMF uses a small set of wheezing components ($K_{w}$), in some cases, the narrowband spectral peaks are divided into several bases of the same matrix $B_{W}$. To clarify this issue, Figure 5 shows the matrix $B_{W}$ obtained for the different examples of MP and PP wheezing described in Section 1. As shown in Figure 5D, the energy of the narrowband spectral patterns, which characterizes that PP wheezing, are divided into two bases $B_{W}\left(1\right)$ and $B_{W}\left(2\right)$ belonging to the matrix $B_{W}$. In both bases, $B_{W}\left(1\right)$ and $B_{W}\left(2\right)$, all narrowband spectral peaks are correctly modeled.

Finally, we propose to obtain the spectral energy distribution ξ(f) (see Equation (18)) from the set of bases that compose the matrix $B_{W}$. This makes it possible to compact the spectral distribution of all narrowband spectral peaks that make up the input MP or PP wheezes to analyze their harmonic structure in Stage II.

(18) $$\xi \left(f\right)=\sum_{k_{w}=1}^{K_{w}}B_{W_{f,k_{w}}},\;f=1,\cdots ,F$$

**Figure 6:**
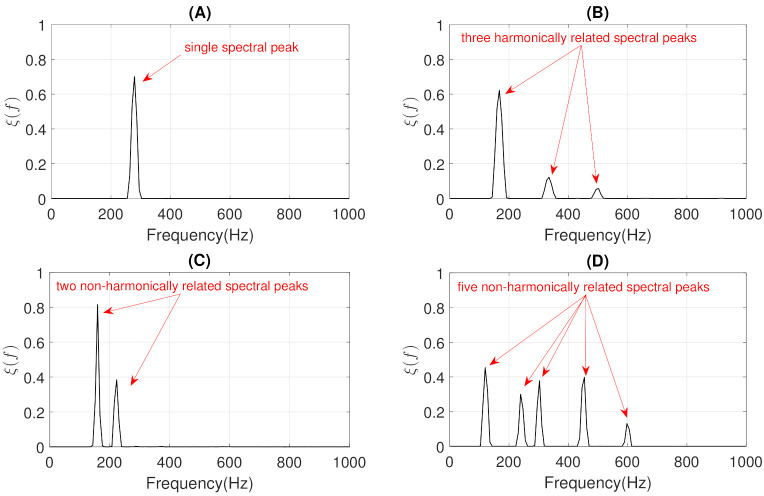
The spectral energy distribution ξ(f) provided by CL-RNMF from the estimated basis matrix $B_{W}$ shown in Figure 5: (**A**) Figure 5A. (**B**) Figure 5B. (**C**) Figure 5C. (**D**) Figure 5D.

Figure 6 shows the spectral energy distribution ξ(f) obtained for the four examples of wheezing shown in Section 1. The pseudocode of this Stage I for the modeling and separation of wheezing spectral patterns based on CL-RNMF is detailed in Algorithm 1.
**Algorithm 1:** CL-RNMF.**Require**: x(t), $K_{r}$, $K_{w}$, α, β, λ, and *M*.ss 1:  Compute the normalized magnitude spectrogram X using Equation (11).ss 2:  Initialize $B_{W}$, $B_{R}$, $A_{W}$, and $A_{R}$ with random non-negative values.ss 3:  Update the estimated wheezing basis matrix $B_{W}$ using Equation (14).ss 4:  Update the estimated respiratory basis matrix $B_{R}$ using Equation (15).ss 5:  Update the estimated wheezing activations matrix $A_{W}$ using Equation (16).ss 6:  Update the estimated respiratory activations matrix $A_{R}$ using Equation (17).ss 7:  Repeat Steps 3–6 until the algorithm converges (or until the maximum number of iterations *M* is reached).ss 8:  Compute the spectral energy distribution ξ(f) from $B_{W}$ using Equation (18).ss **return**
ξ(f)

### 3.3. Stage II: Harmonic Structure Analysis

The goal of this stage is to classify between MP and PP wheezing by analyzing the spectral energy distribution ξ(f) of the different narrowband spectral peaks obtained in the previous stage. Depending on the harmonic structure, wheezing can be classified as MP or PP. Specifically, MP wheezing is composed of a single narrowband spectral peak or the set of harmonically related narrowband spectral peaks. In contrast, PP wheezing is composed of several non-harmonically related narrowband spectral peaks. For this reason, we propose to obtain the number of narrowband spectral peaks η that can be found from ξ(f). Note that the procedure to detect the spectral peaks is a simple task since, as can be seen in Figure 6, the spectral energy distribution ξ(f) from CL-RNMF clearly provides a set of narrowband spectral peaks typically found in wheezing sounds. Once the parameter η is obtained, a preliminary classification of the type of wheezing can be performed as follows,

(19) $$\mathrm{Wheezing}\;\mathrm{category}=\left\{\begin{array}{cc} \mathrm{MP} & \mathrm{if}\;\;\;\eta =1\\ \mathrm{MP}\;\mathrm{or}\;\mathrm{PP} & \mathrm{if}\;\;\;\eta >1 \end{array}\right.$$

**Figure 7:**
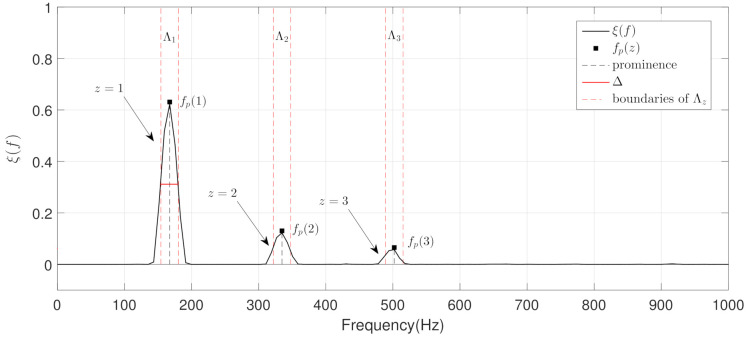
Example of the proposed two-step procedure to classify between MP and PP wheezing when η>1 from the example of MP wheezing shown in Figure 1B. Note that the arrows indicate the narrowband spectral peaks that compose the wheezing. In this case, the wheezing is classified as MP because all spectral peaks are harmonically related.

Wheezing can only be classified as MP when η=1 since a wheezing is composed of a single narrowband spectral peak, as can be seen in Figure 6A. However, a wheezing can be classified as MP or PP when η>1, depending on the harmonic structure that exists between the different narrowband spectral peaks. Specifically, the wheezing is classified as MP if the set of spectral peaks are harmonically related between them. The wheezing is classified as PP if the spectral peaks are not harmonically related between them. In order to perform the classification between MP and PP wheezing in the case of η>1, we propose a two-step procedure, as follows:

- The objective of the first step is to locate, in terms of frequency, all the narrowband spectral peaks detected in the previous Stage I. For this, we propose to locate the most prominent frequency $f_{p}\left(z\right)$ in each spectral peak z=1,…,η. Each value $f_{p}\left(z\right)$ was calculated using the findpeaks function provided by the MATLAB software [74] due to the satisfactory results obtained in several preliminary analyses performed. Figure 7 shows the location $f_{p}\left(z\right)$, in terms of frequency, of each spectral peak for the MP example previously shown in Figure 1B.
- The objective of the second step is to check if the different spectral peaks z=1,…,η are harmonically related or not. We assume that the first spectral peak (z=1) represents the basal peak. Therefore, the wheezing is classified as MP if the rest of spectral peaks (z=2,…,η) are located in the harmonic frequencies (integer multiple) of the basal peak. Otherwise, the wheezing is classified as PP. From the width Δ of the main lobe of the basal peak (z=1) and the value of its most prominent frequency $f_{p}\left(1\right)$, the spectral intervals where the possible harmonic frequencies should be located are calculated as follows,
  (20) $$\Lambda_{z}=\left[zf_{p}\left(1\right)-\left(\Delta /2\right),\;zf_{p}\left(1\right)+\left(\Delta /2\right)\right],\;z=1,\cdots ,\eta$$
  where $\left[i,j\right]$ denotes the spectral interval comprised between the lower limit *i* and the upper limit *j*, in terms of frequency. Specifically, $\Lambda_{1}$ represents the spectral interval associated with the basal peak, and $\Lambda_{z}$ (z=2,⋯,η) corresponds to the spectral intervals where the harmonic frequencies should be located. Note that the width of the main lobe Δ was obtained by positioning the reference line beneath the peak at a vertical distance equal to half the peak prominence [74].

**Figure 8:**
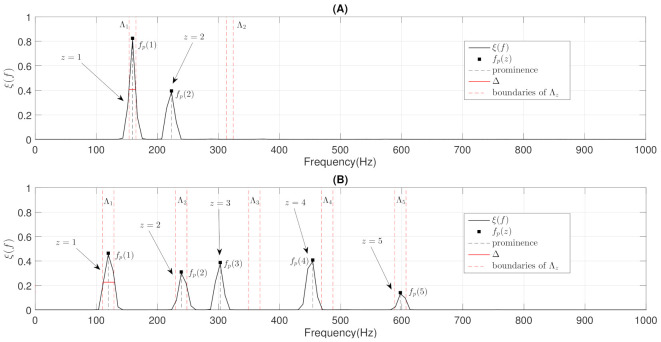
Example of the proposed two-step procedure to classify between MP and PP wheezing when η>1, considering the two examples of PP wheezing shown in Figure 2. (**A**) Two-step procedure applied to the PP wheezing shown in Figure 2A. (**B**) Two-step procedure applied to the PP wheezing shown in Figure 2B. Note that the arrows indicate the narrowband spectral peaks that compose the wheezing. In this case, both wheezing are classified as PP because not all spectral peaks are harmonically related.

Considering the two-step procedure described above, wheezes that are composed of several narrowband spectral peaks (η>1) can be classified as MP or PP as follows,

(21) $$\mathrm{Wheezing}\;\mathrm{category}=\left\{\begin{array}{cc} \mathrm{MP} & \mathrm{if}\;\;\;f_{p}\left(z\right)\subseteq \Lambda_{z},\;z=2,\cdots ,\eta \\ \mathrm{PP} & \mathrm{otherwise} \end{array}\right.$$

where v⊆V denotes that element *v* is contained in the interval *V*. Therefore, when the frequency $f_{p}\left(z\right)$ of all possible harmonic spectral peaks z=2,⋯,η is located in the corresponding spectral intervals $\Lambda_{z}$, the wheezing is classified as MP. Otherwise, wheezing is classified as PP because the narrowband spectral peaks that characterize the wheezing are not harmonically related. This occurs when for the frequency $f_{p}\left(z\right)$, at least one of the possible harmonic spectral peaks is not located in its corresponding spectral intervals $\Lambda_{z}$. Figure 7 shows an example of the procedure described for MP wheezing composed of a basal peak and two harmonics. Figure 8 shows two examples of the procedure described for two PP wheezing with several non-harmonically related spectral peaks. Finally, the pseudocode of this stage for the classification between MP/PP wheezing according to its harmonic structure is detailed in Algorithm 2.
**Algorithm 2:** Harmonic structure analysis. **Require**: ξ(f).  1:  From ξ(f), detect the number η of narrowband spectral peaks.  **if**  η=1  **then**    **return**        Wheezing category = MP  **else**    2:  Locate the frequency $f_{p}\left(z\right)$ in each spectral peak z=1,⋯,η.ssssss    3:  Compute the spectral intervals $\Lambda_{z}$ using Equation (20).    **if**  $f_{p}\left(z\right)\subseteq \Lambda_{z},\;z=2,\cdots ,\eta$  **then**      **return**        Wheezing category = MP     **else**      **return**        Wheezing category = PP     **end if**  **end if**

## 4. Experimental Results and Discussion

### 4.1. Data Collection

As far as the authors’ knowledge, there is no public wheeze database in which wheezing has been labeled as monophonic or polyphonic. For this reason, we received the collaboration of a pneumologist from the University Hospital of Jaén (Spain) to create and label a database according to the wheezing harmonic structure. The database was created by collecting and categorizing a set of recordings from different subjects of the most widely used Internet pulmonary repositories [75,76,77,78,79,80,81,82,83,84,85,86,87]. Specifically, all previous recordings were collected from subjects with CRDs (asthma or COPD). Note that the set of recordings selected for this assessment was only composed of normal respiratory sounds and wheezing sounds.

**Figure 9:**
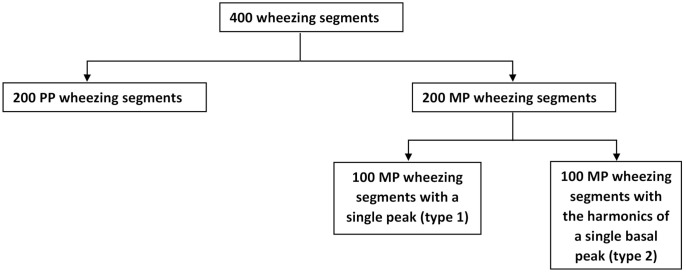
Scheme of the types of wheezing contained in the database.

The type of wheezing (MP or PP) was labeled by the pneumologist by means of an acoustic inspection and a visual verification of the spectrogram considering the harmonic structure that distinguishes both types of wheezing. The database consisted of 200 MP and 200 PP wheezing segments, where the duration of each segment was at least 100 ms, to be consistent with the literature. As mentioned above, MP wheezing can show two different harmonic structures: Type 1, wheezes with a single peak, that is only the fundamental frequency component is active; and Type 2, wheezes with the harmonics of a single basal peak, that is both the fundamental frequency component and its frequencies that are harmonically related are active. Therefore, to guarantee the maximum variability of the MP wheezing, the 200 MP wheezing segments were divided into 100 MP wheezing segments with a single peak and 100 MP wheezing segments with the harmonics of a single basal peak. Note that all segments were independent of each other, since each segment corresponded to a different wheezing from the rest. Finally, all segments in the database were sampled at 4096 Hz and had a length between 100 and 700 ms. Figure 9 shows the classification performed on the database created.

### 4.2. Experimental Setup

To be consistent with the literature, we assumed that wheezing sounds were not active below 100 Hz and above 1000 Hz. For this reason, all segments that compose the database were band-limited from 100 Hz–1000 Hz.

The length of the signal frames was set to N=256 samples (62.5 ms). This frame size was considered large enough to assume a perfect spectral representation of all wheezing frequency components. The overlap between frames was set to 10% (6.25 ms). To obtain the time-frequency representation, windowing with a Hamming window was applied, and the order of the Discrete Fourier Transform (DFT) was set to 2N frequency bins, similar to [9,50]. This DFT size provides a high enough resolution for modeling the spectral patterns of wheezing sounds and was chosen empirically as a trade-off between achieved quality and complexity. Besides, we empirically observed that the reconstruction error converged after 50 iterations, so the maximum number of iterations for the decomposition was equal to M=50.

Finally, note that the performance of the proposed method depends on the initial values with which the basis matrices $B_{W}$, $B_{R}$ and the activation matrices $A_{W}$, $A_{R}$ are initialized. Although the obtained results are not dispersed and keep the same behavior, in order to overcome this issue, we ran the proposed method five times for each segment that composed the database, and the results shown in this paper are averaged values.

### 4.3. Evaluation Metrics

The Accuracy rates (ACC) were used to evaluate the performance of the proposed method, which are commonly used in the field of wheezing classification [7]. In order to provide a fair evaluation of the classification performance obtained by the proposed method and the state-of-the-art algorithms, the following accuracy rates were proposed: (i) $ACC_{G}$ is the ability to correctly classify a wheezing segment as MP or PP; (ii) $ACC_{P}$ represents the ability to correctly classify a wheezing segment as PP; (iii) $ACC_{M}$ corresponds to the ability to correctly classify a wheezing segment as MP; (iv) $ACC_{M1}$ indicates the ability to correctly classify a wheezing segment as MP Type 1; and (v) $ACC_{M2}$ reports the ability to correctly classify a wheezing segment as MP Type 2. The terms used in Equations (22)–(26) are described in Table 1.

(22) $$ACC_{G}=\frac{\left(TP+TM\right)}{\left(TP+TM+FP+FM)\right)}$$

(23) $$ACC_{P}=\frac{TP}{\left(TP+FP\right)}$$

(24) $$ACC_{M}=\frac{TM}{\left(TM+FM\right)}$$

(25) $$ACC_{M1}=\frac{TM1}{\left(TM1+FM1\right)}$$

(26) $$ACC_{M2}=\frac{TM2}{\left(TM2+FM2\right)}$$

### 4.4. State-of-the-Art Method for Comparison

**Figure 10:**
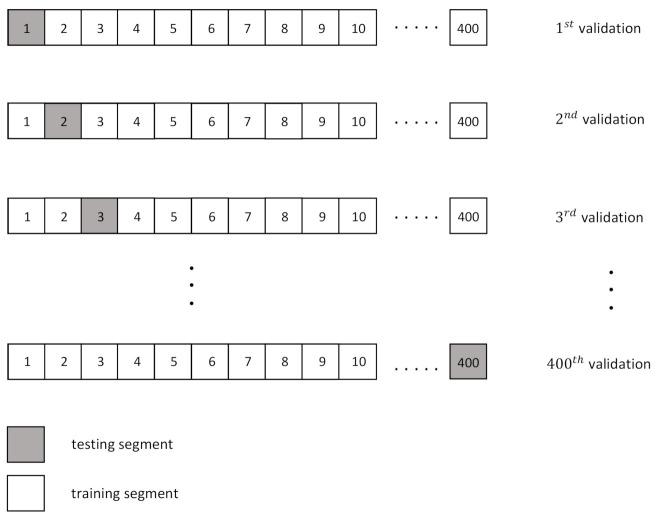
LOO cross-validation scheme for the database described in this paper.

In order to measure the MP/PP classification performance of the proposal, we used the most recent and relevant state-of-the-art algorithm [7], denoted as UPER in this paper. The method UPER was implemented strictly following the instructions specified by the authors in [7]. Firstly, the values of the metric PER were obtained using the 19th parameter set (p=10, q=11, s=7, and J=45) in the RADWT model. Then, three classifiers, Support Vector Machine (SVM) with the Radial Basis Function kernel (RBF kernel), K-Nearest Neighbor (KNN), and Extreme Learning Machine (ELM) were applied to the PER features. The classification performance of UPER was obtained in Leave-One-Out (LOO) cross-validation schemes with the SVM, KNN, and ELM classifiers. Specifically, LOO cross-validation is a particular case of Leave-p-Out (LPO) cross-validation with p=1. Therefore, the LOO scheme involves using one observation as the validation set and the remaining observations as the training set. This is repeated in all ways to cut the database into a validation set of one observation and a training set. Considering the database evaluated in this work (400 segments in total), the LOO cross-validation scheme has 400 possible combinations of validation in which the training set is composed of 399 segments, and only one segment is tested, as can be observed in Figure 10). Results shown in this paper for all classifiers are the average values obtained from the 400 possible validation combinations.

### 4.5. Accuracy Results

In this section, we evaluate the MP/PP classification performance between the proposed method and UPER [7]. A remarkable distinction between the two methods is that the proposed method is completely unsupervised or blind (no training), but the method UPER depends on the training database.

Table 2 shows the MP/PP classification results, in terms of the accuracy rates, evaluating the database described in Section 4.1. Results provided by UPER, considering the three classifier versions (SVM, KNN, and ELM), were obtained by applying an LOO cross-validation scheme as was previously described in Section 4.4. Results report that the proposed method provides the best overall MP/PP classification results compared to UPER considering all evaluated metrics. Focusing on the different accuracy rates, the following can be observed:

- the improvement, in terms of $ACC_{G}$, of the proposed method is about 8.25% UPER (SVM), 12% UPER (KNN), and 10.5% UPER (ELM).
- the improvement, in terms of $ACC_{P}$, of the proposed method is about 4% UPER (SVM), 7.1% UPER (KNN), and 5.5% UPER (ELM).
- the improvement, in terms of $ACC_{M}$, of the proposed method is about 12.5% UPER (SVM), 17% UPER (KNN), and 15.5% UPER (ELM).
- the improvement, in terms of $ACC_{M1}$, of the proposed method is about 5% UPER (SVM), 10% UPER (KNN), and 8% UPER (ELM).
- the improvement, in terms of $ACC_{M2}$, of the proposed method is about 20% UPER (SVM), 24% UPER (KNN), and 23% UPER (ELM).

The main advantage of UPER is that it only uses one feature (PER value) to discriminate between MP and PP wheezing. As shown in Table 2, the SVM classifier obtains the best classification performance in the method UPER. Specifically, the classifier SVM achieves an improvement of 2.25% (KNN) and 1.5% (KNN), in terms of $ACC_{G}$. These results are consistent with those obtained by the authors in [7], confirming that the SVM classifier with the RBF kernel obtains the best classification performance when the number of features (only one PER value) is small [88].

Performing an empirical analysis of the proposed method and UPER, the following observations were extracted:

(i) Due to the time-frequency overlapping problem, normal respiratory sounds often mask wheezing sounds, hiding relevant medical information [5]. While the proposed method (based on CL-RNMF) allows removing as much as possible the acoustic interference from normal respiratory sounds, the method UPER is based on a feature PER obtained from the sub-band energy of the wavelet coefficients, so the presence of normal respiratory sounds interferes in the selection of the optimal sub-bands that really belong to the wheezing components.
(ii) The method UPER has more difficulty in discriminating between PP and MP wheezing composed by a basal peak and its harmonics since it achieves the worst performance in terms of $ACC_{M2}$. The reason is because UPER is based on energy and ignoring the spectral location of the components that model the harmonic behavior of MP wheezing. Results in Table 2 suggest that MP/PP classification based on the spectral location of the harmonic structure as occurs in the proposed method is more reliable than the use of the energy of the wheezing spectral components, as occurs in UPER.

The LOO cross-validation scheme does not show the dependency that classifiers have with the size of the training segments set, since this scheme always uses one segment as the validation set and the remaining segments as the training set. For this reason, we propose to use an LPO cross-validation scheme by varying the size of the training segments set. The LPO scheme requires training and validating the model $C_{p}^{n}$ times, where *n* is the number of segments that compose the database, *p* is the number of validation segments, and $C_{p}^{n}$ is the binomial coefficient. As a result, the associated computational cost can be excessive. In order to overcome this issue, we limited the number of iterations of the LPO scheme to 500. Furthermore, the same number of MP and PP wheezes was selected for both training and validation sets in each iteration. Specifically, we used four LPO schemes: (i) p=80 uses 80% of the total segments as the training set in each iteration; (ii) p=160 uses 60% of the total segments as the training set in each iteration; (iii) p=240 uses 40% of the total segments as the training set in each iteration; and (iv) p=320 uses 20% of the total segments as the training set in each iteration. Considering all the instructions described above, Table 3 shows the MP/PP classification results, in terms of $ACC_{G}$, obtained by UPER using its three classifier versions (SVM, KNN, and ELM) in order to assess its dependence on the training set size. Comparing the LOO scheme with the LPO scheme (p=320), the $ACC_{G}$ reduction of the classification performance is about 7.5% (SVM), 8.25% (KNN), and 6.25% (ELM). Results report that the PER feature allows distinguishing between MP and PP wheezing even when the training set size is reduced. In addition, the ELM classifier shows less dependence on the training database size compared to SVM and KNN.

## 5. Conclusions and Future Work

In this paper, we present a novel Constrained Low-rank Non-negative Matrix Factorization (CL-RNMF) approach to classify monophonic and polyphonic wheezing sounds according to their harmonic structure. The first contribution of this work proposes a CL-RNMF framework that allows extracting the spectral patterns that characterize wheezing sounds with the least possible interference from normal respiratory sounds. Specifically, a low-rank configuration with a reduced number of wheezing bases is presented to compact its frequency components in the least number of bases possible for their posterior analysis. In addition, CL-RNMF uses a set of constraints to model the spectro-temporal behavior of wheezing and normal respiratory sounds. As far as the authors’ knowledge, the non-negative matrix factorization approach has never been applied before to MP/PP wheezing classification. The second contribution analyzes the harmonic structure of the energy distribution from the estimated wheezing spectrogram provided by CL-RNMF to determine the type of wheezing, allowing a more efficient classification based on the location of the wheezing frequency components, rather than the energy of their components.

The most relevant conclusions from the experimental results indicate the following: (i) the proposed method provides the best overall performance related to MP/PP wheezing classification compared to the most relevant method of the state-of-the-art; (ii) unlike most state-of-the-art methods based on classifiers, the proposed method is an unsupervised (blind) approach that does not require any training from wheezing sounds; (iii) the proposed method achieves removing most of the interference from normal respiratory sounds; (iv) specific accuracy rates, $ACC_{M}$ and $ACC_{P}$, obtained by the proposed method seem to suggest the ability of the proposal to classify both monophonic and polyphonic wheezing sounds correctly.

Future work will be focused on the design of new constraints, to be applied in NMF approaches, that improve the modeling of time-frequency respiratory sound events, analyzing different types of adventitious sounds, such as wheezes and crackles. The objective of this future research line is to perform an early detection and classification among the different types of adventitious sounds active in the auscultation process in order to maximize the reliability of the diagnosis issued by the physician in the case of pathologies of lung diseases caused by the appearance of such adventitious sounds.

## Figures and Tables

**Table 1:** Definition of the terms that appear in the metrics detailed in Equations (22)–(26).

Terms	Definitions
TP (True PP)	PP wheezing segments correctly classified
TM (True MP)	MP wheezing segments correctly classified
FP (False PP)	PP wheezing segments misclassified as MP
FM (False MP)	MP wheezing segments misclassified as PP
TM1 (True MP Type 1)	MP Type 1 wheezing segments correctly classified
TM2 (True MP Type 2)	MP Type 2 wheezing segments correctly classified
FM1 (False MP Type 1)	MP Type 1 wheezing segments misclassified as PP
FM2 (False MP Type 2)	MP Type 2 wheezing segments misclassified as PP

**Table 2:** Comparative ACC results between the proposed method and UPER.

Algorithm	${\mathrm{ACC}}_{G}$	${\mathrm{ACC}}_{P}$	${\mathrm{ACC}}_{M}$	${\mathrm{ACC}}_{M1}$	${\mathrm{ACC}}_{M2}$
Proposed Method	**92%**	**91.5%**	**92.5%**	**91%**	**94%**
UPER (SVM) [7]	83.75%	87.5%	80%	86%	74%
UPER (KNN) [7]	80%	84.4%	75.5%	81%	70%
UPER (ELM) [7]	81.5%	86%	77%	83%	71%

**Table 3:** Comparative results, in terms of $ACC_{G}$, between the three classifier versions (SVM, KNN, and ELM) of the method UPER using four Leave-p-Out (LPO) cross-validation schemes.

Scheme	Training Set	Validate Set	SVM	KNN	ELM
LOO	399 (99.75%)	1 (0.25%)	**83.75%**	80%	81.5%
LPO (p=80)	320 (80%)	80 (20%)	**81.5%**	79.25%	80%
LPO (p=160)	240 (60%)	160 (40%)	**80.5%**	77.75%	79.5%
LPO (p=240)	160 (40%)	240 (60%)	**78.25%**	74.75%	77.25%
LPO (p=320)	80 (20%)	320 (80%)	**76.25%**	71.75%	75.25%

## Data Availability

Not applicable.

## Appendix A. Terms of the Multiplicative Update Rules

Here are each of the terms belonging to the multiplicative update rule to obtain the basis matrix $B_{W}$:

(A1) $${\left[\frac{\partial D_{KL}\left(X|\hat{X}\right)}{\partial B_{W}}\right]}^{-}=\left(X⊘\hat{X}\right)A_{W}^{T}$$

(A2) $${\left[\frac{\partial D_{KL}\left(X|\hat{X}\right)}{\partial B_{W}}\right]}^{+}=\left(\left[1\right]A_{W}^{T}\right)$$

(A3) $${\left[\frac{\partial \psi \left(B_{W}\right)}{\partial B_{W}}\right]}_{f,k_{w}}^{-}=\sqrt{F}\left(\frac{B_{W_{f,k_{w}}}\sum_{j=1}^{F}B_{W_{j,k_{w}}}}{{\left(\sum_{j=1}^{F}B_{W_{j,k_{w}}}^{2}\right)}^{\frac{3}{2}}}\right)$$

(A4) $${\left[\frac{\partial \psi \left(B_{W}\right)}{\partial B_{W}}\right]}_{f,k_{w}}^{+}=\frac{1}{\sqrt{\frac{1}{F}\sum_{j=1}^{F}B_{W_{j,k_{w}}}^{2}}}$$

Here are each of the terms belonging to the multiplicative update rule to obtain the basis matrix $B_{R}$:

(A5) $${\left[\frac{\partial D_{KL}\left(X|\hat{X}\right)}{\partial B_{R}}\right]}^{-}=\left(X⊘\hat{X}\right)A_{R}^{T}$$

(A6) $${\left[\frac{\partial D_{KL}\left(X|\hat{X}\right)}{\partial B_{R}}\right]}^{+}=\left(\left[1\right]A_{R}^{T}\right)$$

(A7) $$\begin{array}{cc} {\left[\frac{\partial \phi \left(B_{R}\right)}{\partial B_{R}}\right]}_{f,k_{r}}^{-} & =2F\left(\frac{\left(B_{R_{f-1,k_{r}}}+B_{R_{f+1,k_{r}}}\right)}{\sum_{j=1}^{F}B_{R_{j,k_{r}}}^{2}}\right)+\\ \; & \;+\frac{2FB_{R_{f,k_{r}}}\sum_{j=2}^{F}{\left(B_{R_{j,k_{r}}}-B_{R_{j-1,k_{r}}}\right)}^{2}}{{\left(\sum_{j=1}^{F}B_{R_{j,k_{r}}}^{2}\right)}^{2}} \end{array}$$

(A8) $${\left[\frac{\partial \phi \left(B_{R}\right)}{\partial B_{R}}\right]}_{f,k_{r}}^{+}=\frac{4FB_{R_{f,k_{r}}}}{\sum_{j=1}^{F}B_{R_{j,k_{r}}}^{2}}$$

Here are each of the terms belonging to the multiplicative update rule to obtain the activations matrix $A_{W}$:

(A9) $${\left[\frac{\partial D_{KL}\left(X|\hat{X}\right)}{\partial A_{W}}\right]}^{-}=B_{W}^{T}\left(X⊘\hat{X}\right)$$

(A10) $${\left[\frac{\partial D_{KL}\left(X|\hat{X}\right)}{\partial A_{W}}\right]}^{+}=\left(B_{W}^{T}\left[1\right]\right)$$

(A11) $$\begin{array}{cc} {\left[\frac{\partial \phi \left({\hat{A}}_{W}\right)}{\partial A_{W}}\right]}_{k_{w},t}^{-} & =2T\left(\frac{\left(A_{W_{k_{w},t-1}}+A_{W_{k_{w},t+1}}\right)}{\sum_{i=1}^{T}A_{W_{k_{w},i}}^{2}}\right)+\\ \; & \;+\frac{2TA_{W_{k_{w},t}}\sum_{i=2}^{T}{\left(A_{W_{k_{w},i}}-A_{W_{k_{w},i-1}}\right)}^{2}}{{\left(\sum_{i=1}^{T}A_{W_{k_{w},i}}^{2}\right)}^{2}} \end{array}$$

(A12) $${\left[\frac{\partial \phi \left(A_{W}\right)}{\partial A_{W}}\right]}_{k_{w},t}^{+}=\frac{4TA_{W_{k_{w},t}}}{\sum_{i=1}^{T}A_{W_{k_{w},i}}^{2}}$$

Here are each of the terms belonging to the multiplicative update rule to obtain the activations matrix $A_{R}$:

(A13) $${\left[\frac{\partial D_{KL}\left(X|\hat{X}\right)}{\partial A_{R}}\right]}^{-}=B_{R}^{T}\left(X⊘\hat{X}\right)$$

(A14) $${\left[\frac{\partial D_{KL}\left(X|\hat{X}\right)}{\partial A_{R}}\right]}^{+}=\left(B_{R}^{T}\left[1\right]\right)$$
